# Transcriptome of Male Breast Cancer Matched with Germline Profiling Reveals Novel Molecular Subtypes with Possible Clinical Relevance

**DOI:** 10.3390/cancers13184515

**Published:** 2021-09-08

**Authors:** Veronica Zelli, Valentina Silvestri, Virginia Valentini, Agostino Bucalo, Piera Rizzolo, Ines Zanna, Simonetta Bianchi, Anna Coppa, Giuseppe Giannini, Laura Cortesi, Daniele Calistri, Maria Grazia Tibiletti, Stephen B. Fox, kConFab, Domenico Palli, Laura Ottini

**Affiliations:** 1 Department of Molecular Medicine, Sapienza University of Rome, 00161 Rome, Italy; veronica.zelli@univaq.it (V.Z.); valentina.silvestri@uniroma1.it (V.S.); virginia.valentini@uniroma1.it (V.V.); agostino.bucalo@uniroma1.it (A.B.); piera.rizzolo@fbf-isola.it (P.R.); giuseppe.giannini@uniroma1.it (G.G.); 2 Cancer Risk Factors and Lifestyle Epidemiology Unit, Institute for Cancer Research, Prevention and Clinical Network (ISPRO), 08518 Florence, Italy; i.zanna@ispro.toscana.it (I.Z.); d.palli@ispro.toscana.it (D.P.); 3 Division of Pathological Anatomy, Department of Health Sciences, University of Florence, 08518 Florence, Italy; simonetta.bianchi@unifi.it; 4 Department of Experimental Medicine, Sapienza University of Rome, 00161 Rome, Italy; anna.coppa@uniroma1.it; 5 Department of Oncology and Haematology, University of Modena and Reggio Emilia, 41124 Modena, Italy; hbc@unimore.it; 6 IRCCS Istituto Romagnolo per lo Studio dei Tumori (IRST) “Dino Amadori”, 47014 Meldola, Italy; daniele.calistri@irst.emr.it; 7 Department of Pathology, ASST Settelaghi and Centro di Ricerca per lo Studio dei Tumori Eredo-Familiari, Università dell’Insubria, 21100 Varese, Italy; mariagrazia.tibiletti@asst-settelaghi.it; 8 Department of Pathology, Peter MacCallum Cancer Centre, The University of Melbourne, Melbourne, VIC 3000, Australia; stephen.fox@petermac.org; 9 Sir Peter MacCallum Department of Oncology, The University of Melbourne, Melbourne, VIC 3000, Australia; heather.thorne@petermac.org; 10 Kathleen Cuningham Foundation Consortium for Research into Familial Breast Cancer (kConFab), Research Department, PeterMacCallum Cancer Centre, Melbourne, VIC 3000, Australia

**Keywords:** male breast cancer, transcriptome profiling, germline mutations, *BRCA1/2*, molecular subtypes

## Abstract

**Simple Summary:**

Breast cancer in men is a rare disease; however, morbidity and mortality in male breast cancer (MBC) patients is a serious concern. The identification of specific molecular features in MBC is essential for developing more appropriate and targeted therapeutic strategies for MBC patients. In this study, by transcriptome analysis of 63 MBCs characterized for germline mutations in the most relevant BC susceptibility genes, mainly *BRCA1/2,* we highlighted possible differences in the molecular pathways underlying MBC pathogenesis in relation to germline mutation status. Furthermore, we identified two distinct subgroups of MBCs of clinical relevance, which are characterized by different biological features and prognosis. Overall, our results showed that transcriptome profiling by RNA sequencing is a valuable approach to dissect the molecular heterogeneity of MBC and suggest that the transcriptome matched with germline profiling may lead to the identification of MBC subtypes with possible relevance in the clinical setting, which is a primary step to improve the clinical management of MBC patients.

**Abstract:**

Male breast cancer (MBC) is a rare and understudied disease compared with female BC. About 15% of MBCs are associated with germline mutation in BC susceptibility genes, mainly *BRCA1/2* and *PALB2*. Hereditary MBCs are likely to represent a subgroup of tumors with a peculiar phenotype. Here, we performed a whole transcriptome analysis of MBCs characterized for germline mutations in the most relevant BC susceptibility genes in order to identify molecular subtypes with clinical relevance. A series of 63 MBCs, including 16 *BRCA2,* 6 *BRCA1*, 2 *PALB2*, 1 *RAD50*, and 1 *RAD51D* germline-mutated cases, was analyzed by RNA-sequencing. Differential expression and hierarchical clustering analyses were performed. Module signatures associated with central biological processes involved in breast cancer pathogenesis were also examined. Different transcriptome profiles for genes mainly involved in the cell cycle, DNA damage, and DNA repair pathways emerged between MBCs with and without germline mutations. Unsupervised clustering analysis revealed two distinct subgroups, one of which was characterized by a higher expression of immune response genes, high scores of gene-expression signatures suggestive of aggressive behavior, and worse overall survival. Our results suggest that transcriptome matched with germline profiling may be a valuable approach for the identification and characterization of MBC subtypes with possible relevance in the clinical setting.

## 1. Introduction

Compared with breast cancer (BC) in women, BC in men is a rare and less investigated disease. Inherited mutations in *BRCA1*, *BRCA2,* and *PALB2* predispose to male BC (MBC) and account for up to 15% of all cases [1,2,3]. Additional genes, mainly belonging to or functionally linked to the DNA repair pathways connected to *BRCA1/2,* may also be involved in MBC predisposition [3,4,5,6].

While traditionally thought to be similar to late-onset post-menopausal estrogen/progesterone receptors (ER/PR)-positive female BC [7,8], increasing evidence indicates that MBC may be different, with unique molecular subtypes suggesting gender-specific differences in terms of biological and clinical behavior [9,10,11]. Thus, the identification of specific molecular features in MBC is essential for developing more appropriate clinical management for MBC patients.

In this context, transcriptome profiling is a proven strategy that is able to identify and characterize BC subgroups of biological and clinical relevance. Based on transcriptome profiles, the intrinsic heterogeneity in female BCs sharing the same molecular subtype and/or hormonal receptor status has been dissected [12], and differentially expressed genes and pathways between female BCs associated and non-associated with *BRCA1/2* germline mutations have been also identified [13,14].

To date, only a few studies have been performed to comprehensively characterize MBC transcriptome profiles [15,16,17]. A different transcriptomic landscape in female and male BC has been observed, and the molecular subtypes identified in MBC are not attributable to any of the molecular subtypes identified in female BC [15,16,17].

Overall, little is known about the intrinsic molecular subgroups of MBCs, and no specific data are available with regard to *BRCA1/2* germline mutation status. Previous studies suggest that MBCs associated with *BRCA1/2* germline mutations may be characterized by biological characteristics indicative of aggressive behavior [10,18]. Interestingly, a molecular MBC subgroup characterized by biological aggressiveness and a trend toward worse prognosis was identified by gene expression profiling [16]. 

In this study, we analyzed the transcriptome profiles of MBCs characterized for germline pathogenic variants in the main BC susceptibility genes in order to identify MBC subgroups with possible clinical relevance. The characterization of new, intrinsic molecular subtypes could eventually improve our understanding of the mechanisms underlying MBC pathogenesis and may offer new biomarkers for the clinical management of MBC patients.

## 2. Materials and Methods

### 2.1. Study Population

Cases were selected within the Italian multicenter study on MBC, which comprises samples and data from more than 700 MBCs [3,19], on the basis of availability of tumor samples that could provide an adequate quantity and quality of RNA to carry out molecular analyses and to have an equal ratio of cases with and without germline pathogenic variants (from now on referred to as mutations). 

*BRCA1* and *BRCA2* mutation analysis was performed in the frame of genetic counseling programs at the center of origin for all MBC cases, as previously reported [19]. The majority of cases negative for *BRCA1/2* mutations were retested using Next-Generation Sequencing for germline mutations in 50 cancer-related genes, allowing the identification of mutations in other BC susceptibility genes [3]. To further enrich the series of cases with germline mutations, tumor samples from kConFab were also recruited [20]. A total of 63 MBCs, comprising 26 cases with germline mutations (16 *BRCA2,* 6 *BRCA1*, 2 *PALB2*, 1 *RAD50*, 1 *RAD51D*) and 37 cases without germline mutations were included in this study (Supplementary Table S1). 

All MBCs were characterized for the main clinical–pathologic features, including age at diagnosis, follow-up data, tumor histotype, histologic grade, nodal status, estrogen and progesterone receptors (ER/PR), and HER2 expression, as previously described [19]. The MBC patients analyzed in this study underwent surgery without neoadjuvant therapy administration.

For each MBC case, informed consent was obtained. The study was approved by the Local Ethical Committee (Sapienza University of Rome, Prot. 669/17).

### 2.2. RNA Isolation and Sequencing

RNA from breast tumors was extracted from microdissected FFPE sections using the MiReasy FFPE kit (Qiagen, Hilden, Germany) according to the manufacturer’s instructions. Microdissection was performed to assure that each tumor sample contains at least 70% of tumor cells. RNA quality and quantity were assessed on a 2100 Bioanalyzer instrument (Agilent Technologies, Santa Clara, CA, USA). 

Libraries were prepared using the TruSeq RNA Access Library Prep kit (Illumina, San Diego, CA, USA) following the manufacturer’s protocol. This kit, optimized for sequencing RNA from FFPE tissues, allows the sequence-specific capture of RNA-coding regions. RNA sequencing was performed in paired-end mode (2 × 75 bp) on an Illumina NextSeq platform. 

A bioinformatic pipeline including FastQC for quality control, trimmomatic (version 36) to remove the adapter sequence (if present) and the very short reads (read length < 25 bp), STAR (version 2.5.3a) for alignment on reference homo sapiens hg19 (Ensembl version GRch37), and RSeQC-FPKM for counting reads was used to analyze the data. Overall, a mean of 80% of the reads were mapped to the coding region of the reference human genome, with an average depth of about 20 million reads per sample. 

### 2.3. Data Analysis

#### 2.3.1. Differential Gene Expression Analysis 

For differential expression analysis, R package DESeq2 was used. Batch effects correction was also evaluated but unnecessary. Differentially expressed loci between different groups were assessed based on a *log2 fold change* <−0.58 (down-regulated genes) or >0.58 (up-regulated genes) and an adjusted *p*-value <0.05 (Benjamini–Hochberg FDR correction for multiple testing). 

#### 2.3.2. Gene Enrichment and Pathway-Based Analysis

Gene ontology (GO)-based analysis of the differentially expressed genes was performed using the Database for Annotation, Visualization and Integrated Discovery (DAVID) (http://david.abcc.ncifcrf.gov/; accessed on 22 July 2020) and Enrichr (http://amp.pharm.mssm.edu/Enrichr/; accessed on 22 July 2020) in order to determine the biological relevance of up/down-regulated genes within the considered groups. GO enrichment analysis was mainly performed based on biological process (BP) and molecular function (MF), while pathway analysis was carried out by using the Kyoto Encyclopedia of Genes and Genomes (KEGG). UniProtKB/TrEMBL entry (UP_Keywords) and the Reactome Pathway were also interrogated. A Benjamini–Hochberg-adjusted *p*-value <0.05 was used to filter the terms of GO and pathway analysis. 

Module signatures associated with key biological processes in female and male BC were computed as previously reported [16,21] and used to highlight further differences between MBC subgroups by the Wilcoxon rank-sum test. 

#### 2.3.3. Clustering Analysis

The identification of subgroups with distinct gene expression patterns was also performed using unsupervised hierarchical clustering analysis on the sample correlation matrix, using the 2000 most variable transcripts among tumor samples. Cluster stability was evaluated by multiscale bootstrap resampling with 10,000 bootstrapped datasets using Pvclust [22].

#### 2.3.4. Statistical Analyses 

Clinical–pathologic characteristics between different groups were compared by using the *t*-test and Fisher exact test where appropriate. 

Survival time was calculated from the date of interview to the date of death from any cause (overall survival, OS) or the last follow-up for alive patients, as previously described [23]. The analysis was focused on 10-year survival. OS was estimated using the Kaplan–Meier method, and differences between groups of patients were assessed by the log-rank test.

A *p* value <0.05 was considered statistically significant. All statistical analyses were performed with the R software (www.r-project.org accessed on 22 July 2020) and STATA version 13.1.

## 3. Results

A total of 63 MBCs, comprising 26 cases with germline mutations (germline-mutated MBCs), including 16 *BRCA2*, 6 *BRCA1*, 2 *PALB2*, 1 *RAD50*, 1 *RAD51D*, and 37 cases without germline mutations (non-mutated MBCs) were analyzed. The main clinical–pathologic characteristics of MBC cases are reported in Table 1.

Briefly, mean age at first BC diagnosis was 65.5 years (range 40–91 years), and the mean follow up was 7.3 years (range 1–10). As expected, MBCs were mostly ER and PR positive (95.1% and 93.3%, respectively) and HER2 negative (85.5%). Clinical–pathologic characteristics were compared between germline-mutated and non-mutated cases. As shown in Table 1, germline-mutated MBCs were more likely to have higher histologic grade (*p* = 0.03). Notably, only one triple negative (ER-, PR-, HER2-) MBC was present in our series, and it was a non-mutated case.

Differential expression analysis was firstly performed to characterize the specific transcriptome profiles in MBCs with and without germline mutations. A total of 410 differentially expressed genes, of which 204 were up-regulated and 206 were down-regulated (Supplementary Table S2), emerged in germline-mutated compared with non-mutated MBCs.

Principal component analysis (PCA) and heatmap of correlation coefficients are reported in Supplementary Figure S1. Although a general similarity among all MBC samples was observed (correlation values > 0.6), the PCA plot showed that the set of differentially expressed genes was able to discriminate between germline-mutated and non-mutated MBCs.

Gene set enrichment analysis of differentially expressed genes between germline-mutated and non-mutated MBCs revealed different transcriptome profiles for genes involved in the cell cycle, cell division, translational initiation, and DNA damage and repair pathways (Table 2).

Specifically, based on pathway and GO analysis, key genes in cell cycle regulation (FOXM1 and AURKA) and DNA damage and repair (BARD1, BRIP1 and XRCC2) were significantly up-regulated in germline-mutated MBCs compared with non-mutated MBCs (Supplementary Figure S2A–E).

**Figure 1:**
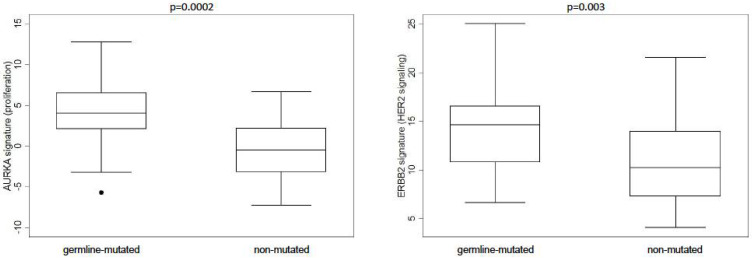
Gene-expression module signatures for key breast cancer biological processes were significantly different between germline-mutated and non-mutated MBCs.

Analysis of gene-expression module signatures showed differences between germline-mutated and non-mutated MBCs for proliferation (AURKA signature, *p* = 0.0002) and HER2 (ERBB2 signature, *p* = 0.003) signaling (Figure 1). In particular, germline-mutated MBCs showed higher proliferation and HER2 signaling module scores compared with non-mutated cases.

**Figure 2:**
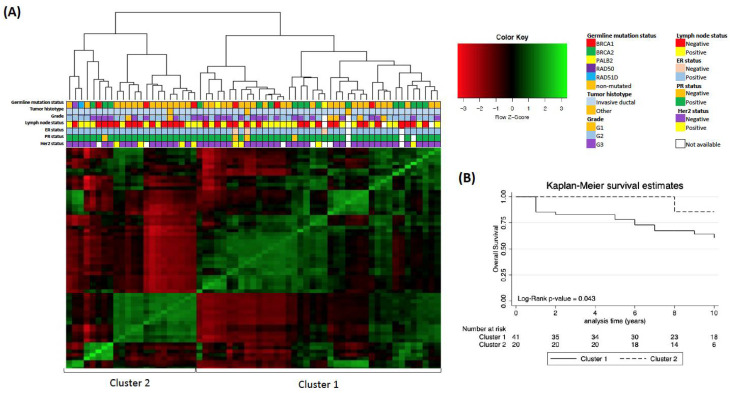
(**A**) Heatmap of the 2000 most variable transcripts among MBC cases showing two clusters (Cluster 1 and Cluster 2). Germline mutation status and pathological characteristics are reported for each case. (**B**) Kaplan–Meier survival analysis suggesting worse overall survival for the cases in Cluster 1 compared with the cases in Cluster 2.

An unsupervised clustering analysis using the 2000 most variable transcripts among all tumor samples was also performed. Using this approach, two distinct MBC clusters emerged (Figure 2A): Cluster 1, which included 41 MBCs, and Cluster 2, which included 22 MBCs (approximately unbiased (AU) probability of 88% and 87%, respectively).

Cluster 1 contained 18 germline-mutated MBCs, including 16 *BRCA1/2*- and two *PALB2*-mutated MBCs, while Cluster 2 contained eight germline-mutated MBCs, including six *BRCA1/2*- and two *RAD*-mutated MBCs. Although Cluster 1 included most germline-mutated cases, the frequency of mutated cases in the two clusters was not significantly different (chi-square *p* = 0.5). No statistically significant differences between Cluster 1 and Cluster 2 cases with regard to clinical–pathologic characteristics emerged (data not shown). Interestingly, the single triple-negative MBC in our series, which is a non-mutated case, clustered in Cluster 1.

Differential expression analysis between the two clusters revealed 431 differentially expressed genes, of which 197 were up-regulated and 234 were down-regulated in Cluster 1 compared with Cluster 2 (Supplementary Table S3). Enrichment analysis highlighted an up-regulation for genes mainly involved in immunity, of both innate and adaptive response, in Cluster 1 (Table 3).

Among relevant genes differentially expressed between the two subgroups, statistically significant different expression levels were observed for NAT1, which was up-regulated in Cluster 2 (*p* = 0.007) (Supplementary Figure S2F).

**Figure 3:**
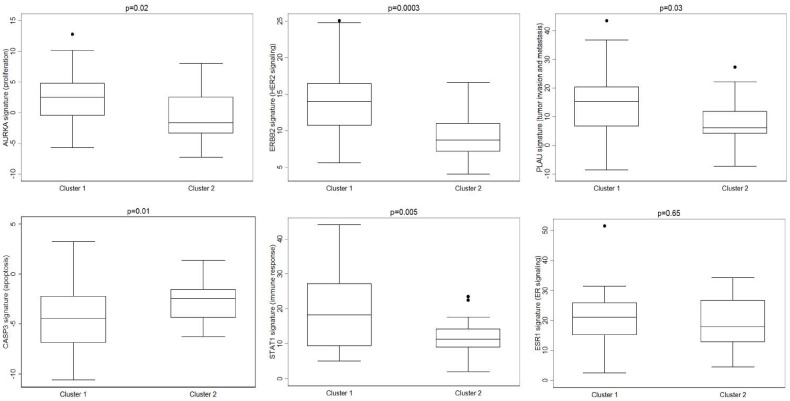
Gene–expression module signatures for key breast cancer biological processes were significantly different between Cluster 1 and Cluster 2 MBCs.

Differences between Cluster 1 and Cluster 2 also emerged for the majority of the gene-expression module signatures evaluated (Figure 3), including proliferation (AURKA signature, *p* = 0.02), HER2 signaling (ERBB2 signature, *p* = 0.0003), invasion and metastasis (PLAU signature, *p* = 0.03), apoptosis (CASP3 signature, *p* = 0.01), and immune response (STAT1 signature, *p* = 0.005). Specifically, Cluster 1 showed higher expression scores compared with Cluster 2 cases for all modules except for apoptosis signaling. No differences between MBC Cluster 1 and Cluster 2 and ER signaling emerged (Figure 3).

The Kaplan–Meier survival estimate, which was performed to evaluate the OS probability, showed a worse outcome for MBC cases in Cluster 1 compared with the cases in Cluster 2 (log-rank test *p* = 0.043) (Figure 2B).

## 4. Discussion

In order to identify molecular subtypes with possible clinical relevance, we investigated the transcriptome profiles of a series of MBC cases all characterized for germline mutations in the most relevant BC susceptibility genes [3]. To the best of our knowledge, this is the first gene expression study performed in MBC using RNA sequencing, which is an approach demonstrated to provide considerable advantages in terms of number of identifiable differentially modulated transcripts [24,25,26]. Given that MBCs associated with germline mutations in *BRCA* are likely to represent a subgroup with a peculiar phenotype, analyzing transcriptome profiles by genetic factors could provide additional insights to better stratify patients eligible for a personalized management.

Differential expression analysis in MBCs with and without germline mutations showed a statistically significant up-regulation of genes involved in cell cycle regulatory pathways, such as *FOXM1* and *AURKA*, in germline-mutated compared with non-mutated MBCs. In line with these findings, a significant overexpression of cell cycle-related genes, in particular *FOXM1*, has been observed in *BRCA*-mutated compared with non-mutated female BC cases [14]. *FOXM1* plays a central role in the regulation of the cell cycle, and the up-regulation of *FOXM1* in *BRCA*-mutated BCs has been suggested as a result of response to DNA damages [14]. Furthermore, *FOXM1* overexpression was identified as an independent marker of poor prognosis in MBC [27]. Overall, our findings together with these data point to FOXM1 as a key regulator and potential prognostic factor in MBC, particularly for germline-mutated cases.

Similarly, a higher frequency of *AURKA* amplification, a key regulator of the mitotic cell division process, has been reported in tumors from *BRCA*-mutated female BC cases compared to non-mutated cases, suggesting a possible cooperation between *AURKA* overexpression and *BRCA* inactivation in tumor development and progression [28]. Consistent with the high expression of the *AURKA* gene in germline-mutated cases, we found a statistically significant difference in the *AURKA* (i.e., proliferation) gene-expression signature between germline-mutated and non-mutated MBCs. Overall, these findings add to our previous data showing that *BRCA*-associated MBCs may exhibit pathological features suggestive of biological aggressiveness [10].

The up-regulation of genes belonging to DNA damage and repair pathways also emerged in germline-mutated compared with non-mutated MBCs. These results may suggest a possible compensatory mechanism in germline-mutated tumors. The activation of an alternative DNA repair mechanism in tumors with defects in homologous recombination genes, such as those found mutated in this MBC series, has provided the rationale for the development of targeted therapy using PARP inhibitors (PARPi) [29]. Overall, our results may indicate distinct underlying molecular pathways in MBCs associated with germline mutations in homologous recombination genes, which may influence the managements of this subset of patients [30]. Studies on larger series of germline-mutated MBC cases, possibly characterized for somatic alterations, are needed to further shed light on molecular mechanisms underlying mutated tumors and on potentially actionable molecular targets.

To further characterize MBC subgroups without any *a priori* classification, an alternative approach based on unsupervised clustering was used. This analysis showed two distinct MBC subgroups (Cluster 1 and Cluster 2), which are not attributable to known clinical–pathologic features.

Immune response emerged as the most relevant process able to discriminate Cluster 1 and Cluster 2 at the transcriptional level. Avoiding the immune destruction and the tumor-promoting inflammation are currently widely accepted as hallmarks of cancer development, and understanding the composition and interaction between cancer and the immune system in the tumor microenvironment is essential for the clinical applicability of immunotherapy strategies, which is a cutting-edge cancer treatment [31]. To date, little is known about the role of the immune system in MBC; thus, these findings are open to further analysis.

The investigation of key biological processes using gene module scores showed that Cluster 1 tumors were associated with higher scores of immune responses, tumor invasion and metastasis, proliferation, and HER2 signaling modules as well as a lower score of apoptosis module compared with MBCs in Cluster 2. Notably, MBC cases in Cluster 1 displayed worse clinical outcome. Overall, these findings indicate that MBCs in Cluster 1 may be more aggressive than those in Cluster 2. Furthermore, germline-mutated MBCs were associated with higher proliferation and HER2 signaling module scores, suggesting that Cluster 1 cases may share some biological features of aggressiveness with germline-mutated cases.

A previous study by Johansson et al. showed two MBC subgroups, termed luminal M1 and M2, which were characterized by different biological features and clinical outcome [16]. In particular, the luminal M1 subgroup was characterized by a trend toward a worse prognosis and down-regulation of *NAT1*, which is a gene with possible prognostic and predictive significance in MBC [16]. In this study, *NAT1* was down-regulated in Cluster 1. It is noteworthy that high *NAT1* expression was reported to have a role in ER+ female BC as a predictive marker of response to tamoxifen [32], which is the most often used first antiestrogen drug in MBC patients eligible for endocrine therapy [33].

Despite some similarities, the subgroups identified by Johansson et al. and by this study did not exactly overlap. The investigation of key biological processes using gene module scores showed that similar to Luminal M1 tumors, Cluster 1 tumors were associated with tumor invasion and metastasis, proliferation, and HER2 signaling modules. However, in our study, the ER signaling module did not emerge as a discriminating process between subgroups as in Johansson et al. [16]. These differences may be due to the different methodology used in the two studies (microarray vs. RNA-seq), and also to the enrichment for germline-mutated cases in our series, which may have influenced the unsupervised clustering.

Overall, our results indicate that although phenotypically similar, e.g., ER/PR+ and HER2-, MBCs may display different molecular subtypes that may help in identifying subgroups of patients that may benefit from specific treatment approaches besides endocrine therapy, highlighting the need to include male breast patients in clinical trials, as recently suggested by the FDA [34].

We can speculate that germline-mutated and Cluster 1 MBC cases, characterized by the activation of the cell cycle pathway, might benefit from the use of CDK4/6 inhibitors in combination with endocrine therapy, which is a treatment approved for MBC patients with ER/PR-positive, HER2-negative advanced or metastatic cancer [33,35].

Both germline-mutated and Cluster 1 MBC cases were characterized by a high HER2 score, suggesting that the HER2 pathway may be active; thus, they might benefit from treatment with trastuzumab, as previously reported in female BC [36,37].

Furthermore, germline-mutated MBC cases within Cluster 1, characterized by high immunity pathway expression, might be possible candidates for a synergistic use of PARPi-based therapies and immunotherapy, which is a novel therapeutic strategy proven to be effective in several solid tumors, including breast cancer, and particularly in cases with mutations in homologous recombination genes [38].

Given the rarity of MBC and the low frequency of germline mutations [3], the number of cases analysed in this study is comparable to that of previous studies on gene expression profiling in MBC [15,16]. Unfortunately, some clinical pathologic data were missing, and data on adjuvant therapy were not available.

Very recently, transcriptomic gene risk signatures obtained from several multiparametric tests, including Prosigna, MammaPrint, and Oncotype Dx, have been shown to provide comparable prognostic information in male and female BC [8]. On the other hand, a previous study based on the comparison of the somatic landscape in male and female BCs highlighted the molecular uniqueness and heterogeneity of MBC genetics, for which specific clinical management should be required. [39]. In this context, additional molecular studies on well-characterized larger series, enriched for germline-mutated cases, are needed to validate our results and to correlate the identified MBC molecular subgroups with somatic alterations, treatment, and prognosis.

## 5. Conclusions

In conclusion, our results indicate that germline mutation status could impact on MBC transcriptome profiles, defining subgroups that may be driven by different underlying molecular pathways. Overall, transcriptome profiling by RNA sequencing might be a valuable approach to dissect the molecular heterogeneity of MBC and to identify MBC subgroups of biological and clinical relevance, which is a primary step to improve the clinical management of MBC patients.

## Figures and Tables

**Table 1:** Clinical–pathologic characteristics of the 63 MBCs analyzed in this study and comparison between germline-mutated and non-mutated cases.

Characteristic ^1^	MBCs (N. 63)		Germline- Mutated MBCs (N. 26)		Non-Mutated MBCs (N. 37)		*p*-Value ^2^
	N	%	N	%	N	%	
**Mean age at diagnosis ± SD (range)**	65.5 ± 11.0 (40–91)		65.0 ± 12.3 (43–85)		65.7 ± 10.1 (40–91)		0.8
**Mean follow-up, years ± SD (range)**	7.3 ± 3.1 (1–10)		6.8 ± 3.2 (1–10)		7.6 ± 3.2 (1–10)		0.3
**Tumor histotype**							
Invasive ductal carcinoma	60	95.2	25	100.0	35	92.1	
Other	3	4.8	0	0.0	3	7.9	0.3
**Histologic grade**							
1	6	9.8	0	0.0	6	16.2	
2	29	47.6	10	41.7	19	51.4	
3	26	42.6	14	58.3	12	32.4	**0.03**
**Lymph node status**							
Negative	31	52.5	13	54.2	18	51.4	
Positive	28	47.5	11	45.8	17	48.6	1.0
**ER status**							
Negative	3	4.9	2	8.3	1	2.7	
Positive	58	95.1	22	91.7	36	97.3	0.6
**PR status**							
Negative	4	6.7	3	13.0	1	2.7	
Positive	56	93.3	20	87.0	36	97.3	0.2
**HER2 status**							
Negative	47	85.5	17	81.0	30	88.2	
Positive	8	14.5	4	19.0	4	11.8	0.5

^1^ Some data for each pathologic characteristic are not available. ^2^ *p*-value < 0.05 in bold text.

**Table 2:** Enrichment analysis of up-regulated (N = 204) and down-regulated (N = 206) genes in germline-mutated MBCs compared with non-mutated MBCs. Only the terms considered as the most representative and interesting among the significant ones are reported.

Category	Term	Expression ^1^	Count	Genes	*p*-Value
**UP_KEYWORDS**	Cell cycle	↑	22	KIFC1, PARD6B, DBF4B, TICRR, DLGAP5, FOXM1, RBL1, KNTC1, KIF18B, AURKA, CENPE, UBE2C, SYCP2, CDKN3, CCNE2, CDC45, NCAPG, HJURP, CIT, CCNA2, ASPM, MELK	4.70 × 10^−7^
**UP_KEYWORDS**	Cell division	↑	13	KIFC1, PARD6B, KNTC1, KIF18B, CENPE, AURKA, UBE2C, SYCP2, CCNE2, NCAPG, CIT, CCNA2, ASPM	8.00 × 10^−4^
**UP_KEYWORDS**	Mitosis	↑	10	KIFC1, NCAPG, KNTC1, KIF18B, CENPE, AURKA, UBE2C, CIT, CCNA2, ASPM	0.002
**KEGG_PATHWAY**	hsa03010:Ribosome	↓	9	RPS25, RPS17, RPL34, RPLP1, RPL26, RPS9, RPL24, RSL24D1, RPL4	0.003
**GOTERM_BP_DIRECT**	GO:0051301—cell division	↑	13	KIF14, KIFC1, PARD6B, KNTC1, KIF18B, CENPE, AURKA, UBE2C, SYCP2, CCNE2, NCAPG, CCNA2, TUBA1C	0.005
**GOTERM_BP_DIRECT**	GO:0006413~translational initiation	↓	9	RPS25, RPS17, RPL34, RPLP1, RPL26, EIF3L, RPS9, RPL24, RPL4	0.009
**UP_KEYWORDS**	DNA damage	↑	9	XRCC2, TICRR, FOXM1, BRIP1, ATAD5, RAD54B, POLQ, RAD54L, BARD1	0.032
**GOTERM_CC_DIRECT**	GO:0070062—extracellular exosome	↓	40	KRT6C, NAMPT, C3, CSF1, SORL1, HEXB, CLU, ITM2B, RPS25, AZGP1, GPM6A, SERINC1, RPL34, RPLP1, FAT2, HSPA6, IGKV1D-12, LTF, RPL4, PRKACB, PDGFD, ARL6IP5, MUC13, RHOBTB3, PLAT, FLOT2, TMC5, RPL26, RPS9, RPL24, ENDOD1, SERPINI1, NUCB1, GNB2, RPS17, NUCB2, CYBRD1, CYFIP2, CA2, LRP2	0.039
**GOTERM_CC_DIRECT**	GO:0005783—endoplasmic reticulum	↓	18	CAST, EEF1B2, CAMLG, CLU, SORL1, APH1B, VASH1, LRPAP1, FMO5, EPHA4, BRINP1, NUCB2, BCAP29, RAB38, ANO7, LRP2, KCNQ1, ARL6IP5	0.040
**UP_KEYWORDS**	DNA repair	↑	8	XRCC2, TICRR, FOXM1, BRIP1, RAD54B, POLQ, RAD54L, BARD1	0.041
**KEGG_PATHWAY**	hsa04110:Cell cycle	↑	6	CCNE2, CDC45, RBL1, TTK, ESPL1, CCNA2	0.044

^1^ ↑ up-regulated genes, ↓ down-regulated genes.

**Table 3:** Enrichment analysis of up-regulated (N = 197) and down-regulated (N = 234) genes in Cluster 1 compared with Cluster 2 MBCs. Only terms considered as the most representative and interesting among the significant ones are reported.

Category	Term	Expression ^1^	Count	Genes	*p*-Value
UP_KEYWORDS	Immunity	↑	16	MICB, S100A8, IFITM2, GSDMD, IDO2, C4BPB, LY9, APOBEC3H, IGKV1-12, TLR9, IGHV3-11, NUDCD1, IGHV3-23, IGKV1D-39, IGKV3-20, IGHV3-13	0.002
UP_KEYWORDS	Immunoglobulin domain	↑	16	IGHG1, MICB, IL18RAP, MPZL2, LY9, PIGR, SIRPB1, SIGIRR, IGHV3-11, LINGO1, IGSF5, IGHV3-23, IGKV1D-39, IGKV3-20, IGHV3-13, LAG3	0.003
GOTERM_MF_DIRECT	GO:0003823~antigen binding	↑	8	IGHG1, IGHV3-11, MICB, IGHV3-23, IGKV1D-39, IGKV3-20, IGHV3-13, LAG3	0.005
REACTOME_PATHWAY	R-HSA-166663:R-HSA-166663 Initial triggering of complement	↑	6	IGHG1, IGHV3-11, IGHV3-23, IGKV1D-39, IGKV3-20, IGHV3-13	0.015
REACTOME_PATHWAY	R-HSA-2029481:R-HSA-2029481 FCGR activation	↑	6	IGHG1, IGHV3-11, IGHV3-23, IGKV1D-39, IGKV3-20, IGHV3-13	0.017
REACTOME_PATHWAY	R-HSA-2029485:R-HSA-2029485 Role of phospholipids in phagocytosis	↑	6	IGHG1, IGHV3-11, IGHV3-23, IGKV1D-39, IGKV3-20, IGHV3-13	0.019
REACTOME_PATHWAY	R-HSA-198933:R-HSA-198933 Immunoregulatory interactions between a Lymphoid and a non-Lymphoid cell	↑	8	KLRB1, IGHV3-11, MICB, IGHV3-23, IGKV1D-39, IGKV3-20, IGHV3-13, HCST	0.019
REACTOME_PATHWAY	R-HSA-173623:R-HSA-173623 Classical antibody-mediated complement activation	↑	6	IGHG1, IGHV3-11, IGHV3-23, IGKV1D-39, IGKV3-20, IGHV3-13	0.022
UP_KEYWORDS	Immunoglobulin V region	↑	5	IGHV3-11, IGHV3-23, IGKV1D-39, IGKV3-20, IGHV3-13	0.022
UP_KEYWORDS	Adaptive immunity	↑	8	IGHV3-11, MICB, IGHV3-23, IGKV1D-39, IGKV3-20, LY9, IGKV1-12, IGHV3-13	0.025
REACTOME_PATHWAY	R-HSA-5690714:R-HSA-5690714 CD22 mediated BCR regulation	↑	5	IGHV3-11, IGHV3-23, IGKV1D-39, IGKV3-20, IGHV3-13	0.035
REACTOME_PATHWAY	R-HSA-2454202:R-HSA-2454202 Fc epsilon receptor (FCERI) signaling	↑	5	IGHV3-11, IGHV3-23, IGKV1D-39, IGKV3-20, IGHV3-13	0.035

^1^ ↑ up-regulated genes, ↓ down-regulated genes.

## Data Availability

The data that support the findings of this study are available from the corresponding author upon reasonable request.

## Supplementary Materials

The following are available online at https://www.mdpi.com/article/10.3390/cancers13184515/s1, Figure S1: PCA plots and heatmaps of correlation coefficients by using all transcripts (A and C respectively) and the set of differentially expressed genes between germline-mutated and non-mutated MBCs (B and D respectively), Figure S2: (A–E) Boxplots showing the expression, as normalized counts, of relevant differentially expressed genes in MBCs with and without germline mutations: BARD1, BRIP1, XRCC2, FOXM1, AURKA. (F) Boxplots showing the expression, as normalized counts, of NAT1 gene in Cluster 1 compared to Cluster 2. The Benjamini–Hochberg FDR adjusted *p*-value is reported, Table S1: Pathogenic mutations identified in the analyzed MBC series, Table S2: List and functional annotation of the 410 differentially expressed genes in germline-mutated compared with non-mutated MBCs, Table S3: List and functional annotation of the 431 differentially expressed genes in Cluster 1 compared with Cluster 2 MBCs.
