# Supplementary material for: Transcriptome of Male Breast Cancer Matched with Germline Profiling Reveals Novel Molecular Subtypes with Possible Clinical Relevance
Source: Cancers (Basel). 2021 Sep 8;13(18):4515. doi: 10.3390/cancers13184515 (PMC8469418; doi:10.3390/cancers13184515)
Supplement: Supplementary file 1 [file cancers-13-04515-s001.zip › Table S1.pdf]

Supplementary Table S1. Pathogenic mutations identified in the analyzed MBC series.

ID case	Gene	Variant type	Nucleotide change*	Protein change
#17	*BRCA1*	Large deletion	Del exons 21_24	-
#2	*BRCA1*	Frameshift	c.3228_3229del	p.Gly1077AlafsTer8
#31	*BRCA1*	Frameshift	c.2679_2682del	p.Lys893AsnfsTer106
#60	*BRCA1*	Frameshift	c.4964_4982del	p.Ser1655TyrfsTer16
#63	*BRCA1*	Large deletion	Del exons 1_23	-
#66	*BRCA1*	Stop gain	c.5266dup	p.Gln1756ProfsTer74
#14	*BRCA2*	Splice acceptor variant	c.632-2A>G	-
#18	*BRCA2*	Stop gain	c.9382C>T	p.Arg3128Ter
#19	*BRCA2*	Frameshift	c.6626_6627del	p.Ile2209ArgfsTer15
#21	*BRCA2*	Stop gain	c.7480C>T	p.Arg2494Ter
#25	*BRCA2*	Frameshift	c.5796_5797del	p.His1932GlnfsTer12
#26	*BRCA2*	Large deletion	Del exons 14_16	-
#28	*BRCA2*	Stop gain	c.9382C>T	p.Arg3128Ter
#29	*BRCA2*	Frameshift	c.2760del	p.Ile921PhefsTer39
#30	*BRCA2*	Frameshift	c.5722_5723del	p.Leu1908ArgfsTer2
#36	*BRCA2*	Stop gain	c.289G>T	p.Glu97Ter
#61	*BRCA2*	Stop gain	c.6037A>T	p.Lys2013Ter
#62	*BRCA2*	Frameshift	c.7940_7941insC	p.Ser2648LysfsTer14
#64	*BRCA2*	Frameshift	c.6743_6755del	p.His2248LeufsTer28
#65	*BRCA2*	Frameshift	c.2760del	p.Ile921PhefsTer39
#67	*BRCA2*	Frameshift	c.8247_8248del	p.Lys2750AspfsTer13
#70	*BRCA2*	Frameshift	c.9098_9099insA	p.Gln3034fs
#68	*PALB2*	Frameshift	c.2167_2168delAT	p.Met723ValfsTer21
#71	*PALB2*	Stop gain	c.1984A>T	p.Lys662Ter
#69	*RAD50*	Frameshift	c.1238_1241delAACT	p.Gln413ArgfsTer2
#56	*RAD51D*	Frameshift	c.293delA	p.Asp98ValfsTer25

*Based on *BRCA1* NM_007294.3, *BRCA2* NM_000059.4, *PALB2* NM_024675.3, *RAD50* NM_005732.3, *RAD51D* NM_001142571.1
