# Supplementary figures and images for: Transcriptome of Male Breast Cancer Matched with Germline Profiling Reveals Novel Molecular Subtypes with Possible Clinical Relevance
Source: Cancers (Basel). 2021 Sep 8;13(18):4515. doi: 10.3390/cancers13184515 (PMC8469418; doi:10.3390/cancers13184515)

(A)

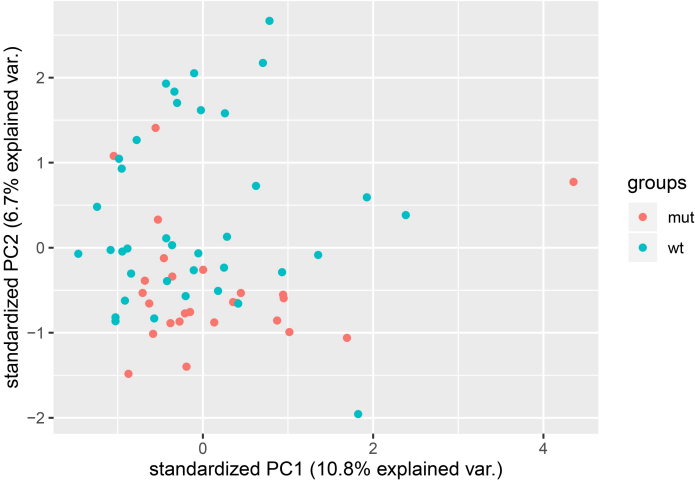

(B)

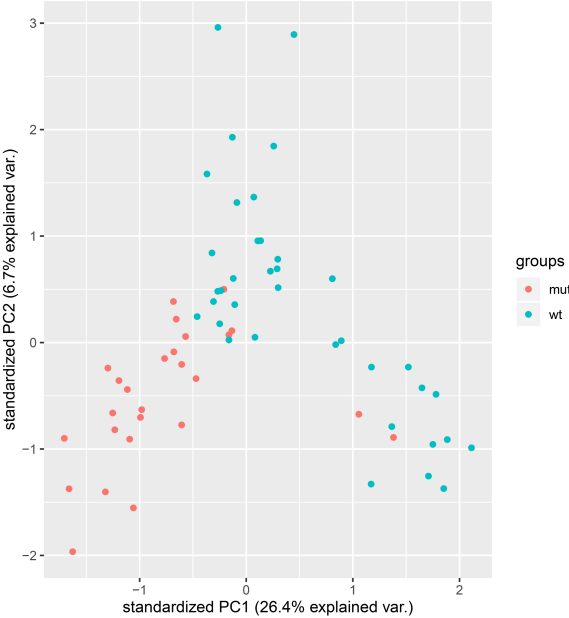

(C)

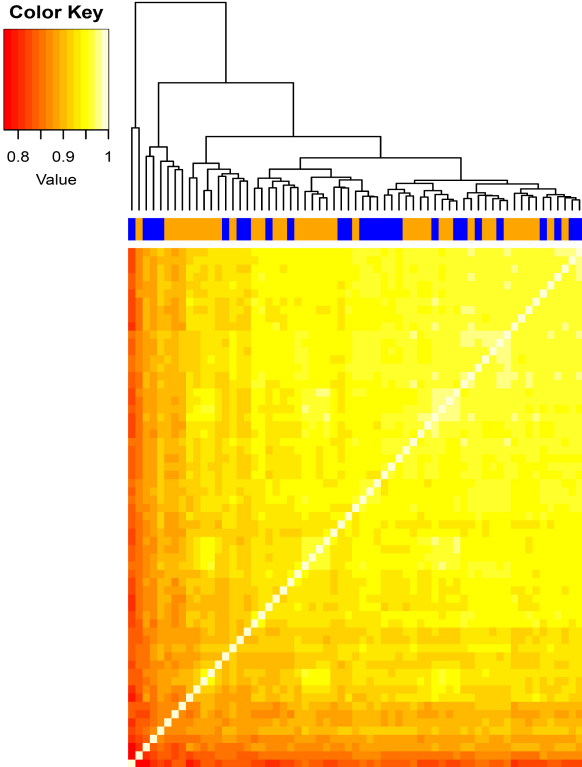

(D)

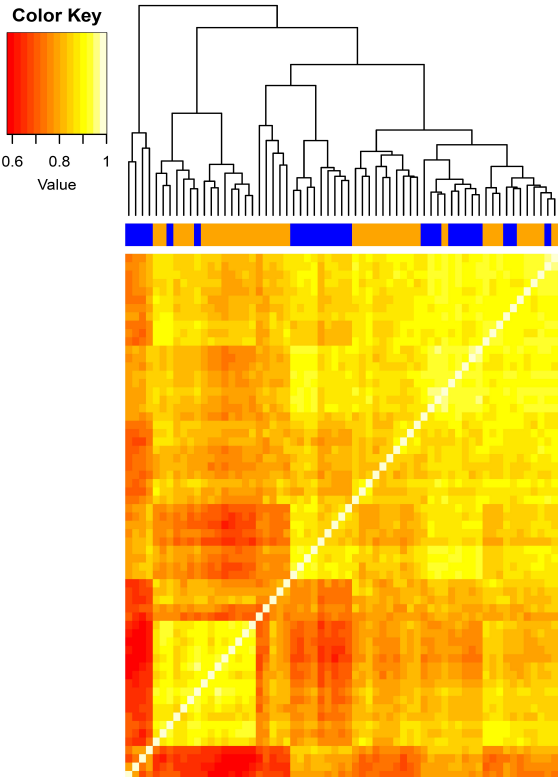

Supplement: Supplementary file 1 [file cancers-13-04515-s001.zip › Figure S1.pdf]

**(A)****BARD1** $p=0.015$ 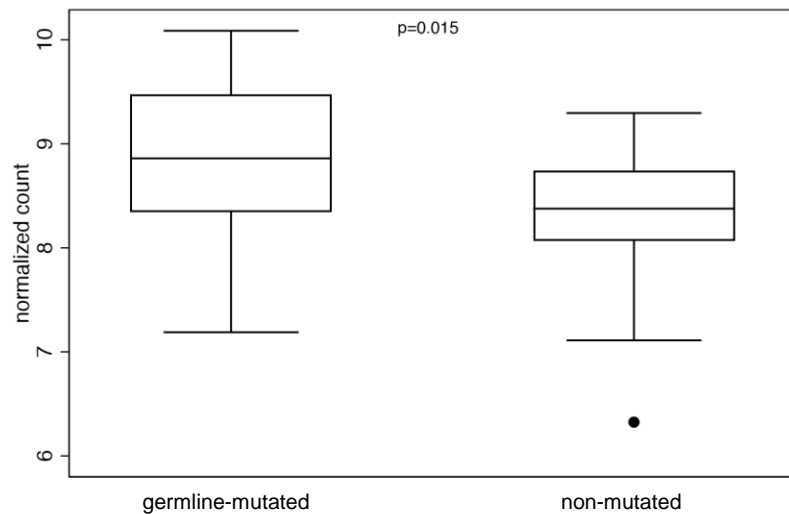**(B)****BRIP1** $p=0.00001$ 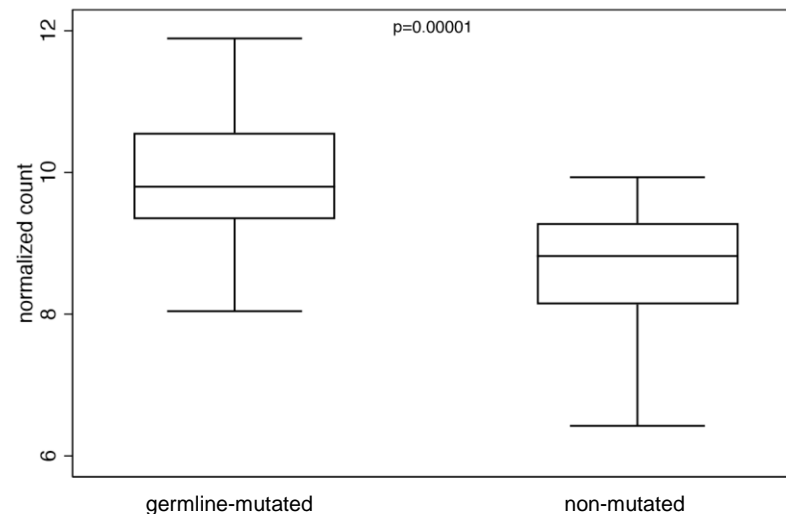**(C)****XRCC2** $p=0.045$ 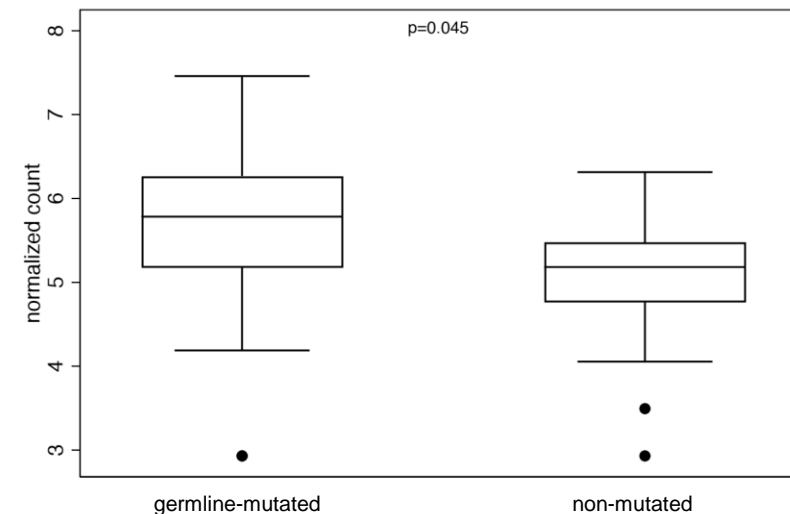**(D)****FOXO1** $p=0.016$ 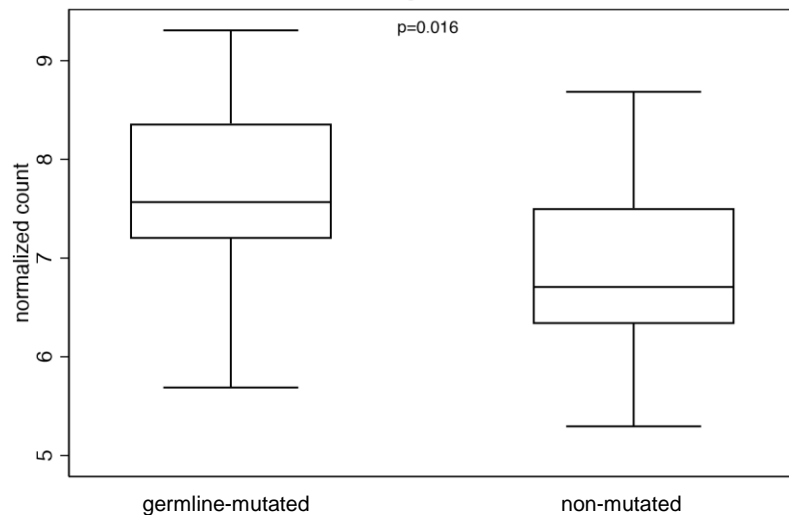**(E)****AURKA** $p=0.003$ 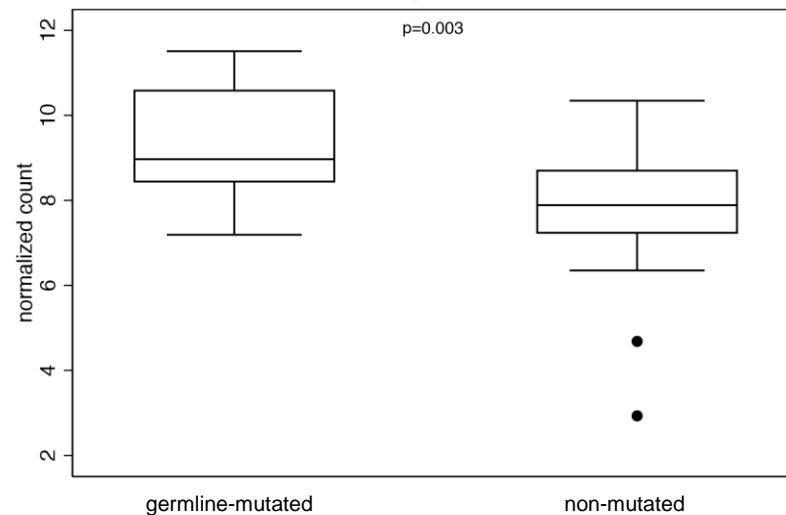**(F)****NAT1** $p=0.007$ 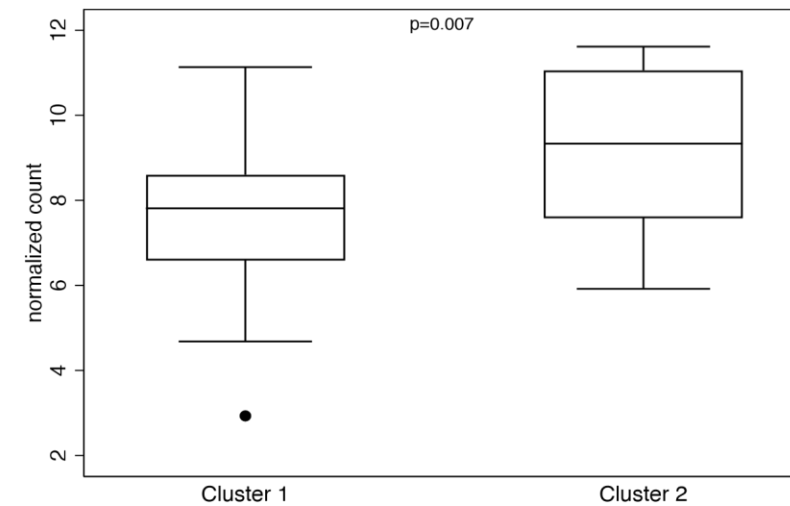

Supplement: Supplementary file 1 [file cancers-13-04515-s001.zip › FigureS2.pdf]
